# Transcriptomes in peripheral blood of young females with temporomandibular joint osteoarthritis

**DOI:** 10.1038/s41598-021-88275-8

**Published:** 2021-04-23

**Authors:** Jeong-Hyun Kang

**Affiliations:** Clinic of Oral Medicine and Orofacial Pain, Institute of Oral Health Science, Ajou University School of Medicine, 164, Worldcup-ro, Yeongtong-gu, Suwon, Gyeonggi-do 16499 Republic of Korea

**Keywords:** Diseases, Medical research, Molecular medicine, Pathogenesis

## Abstract

This study aimed to investigate immune-related pathophysiology of the temporomandibular joint (TMJ) osteoarthritis (OA) in young females by analyzing transcriptional profiles of peripheral blood mononuclear cells. The RNA-sequencing (RNA-seq) was conducted on 24 young females with TMJ OA (mean age 19.3 ± 3.1 years) (RNAOA) and 11 age and sex matched healthy controls (mean age 20.5 ± 3.7 years) (CON). RNA-seq datasets were analyzed to identify genes, pathways, and regulatory networks of those which were involved in the development of TMJ OA. RNA-seq data analysis revealed 41 differentially expressed genes (DEGs) between RNAOA and CON. A total of 16 gene ontology (GO) terms including three molecular and 13 biological terms were annotated via the GO function of molecular function and biological process. Through ingenuity pathway analysis (IPA), 21 annotated categories of diseases and functions were identified. There were six hub genes which showed significant results in both GO enrichment analysis and IPA, namely HLA-C, HLA-F, CXCL8, IL11RA, IL13RA1, and FCGR3B. The young females with TMJ OA showed alterations of the genes related to immune function in the blood and some of changes may reflect inflammation, autoimmunity, and abnormal T cell functions.

## Introduction

Osteoarthritis (OA) is characterized by degradation of components of the extracellular matrix within the articular cartilage and simultaneous remodeling of the underlying subchondral bone, in association with low inflammatory changes^1^. The epidemiology of OA of the temporomandibular joint (TMJ) is different from that of OA of the hand, knee, or hip joint, the incidence of which is associated with aging. Previous reports have already described female preponderance and early onset of the disease, especially from the pubertal phases to the age of early 20 s^2,3^. The pathophysiology of the TMJ OA is multifactorial and complex, which includes diverse etiological factors such as prolonged parafunctional habits, abnormal occlusal relationship, sustained masticatory muscle tension, and hormonal imbalance^4,5^. Given the early onset of the condition, TMJ OA cannot be considered to be just a simple degenerative disease such as the OA of other joints associated with the aging process, so other etiological factors could be assumed. Nevertheless, the clear pathophysiology of the TMJ OA, particularly in young patients has not yet been elucidated so far.

Many emerging evidences have demonstrated the role of immune modulation mechanisms in the development and progression of OA^6–13^. These processes involved immune-modulating agents, in both innate and adaptive compartments such as cytokines, chemokines, T cells, and B cells^6–13^. TMJ OA has been considered to be a low inflammatory arthritic condition and mainly depends on inflammation and elevated levels of inflammatory mediators and cytokines in the TMJ synovial fluid^14–17^. Even though one report suggested the possibility of involvement of systemic immune dysfunction in occurrence of TMJ OA^18^, the influences of systemic immune function and composition of immune cells in the peripheral blood on incidence and progression of TMJ OA have not been fully clarified.

The RNA sequencing (RNA-seq) technology has been utilized as a powerful tool to discover potential molecular mechanisms or therapeutic targets in various diseases^19^. However, to the best of the knowledge, there are yet no studies which adopted RNA-seq technology to patients with TMJ OA. Therefore, the molecular and genetic background of TMJ OA remains obscure, and the clinical treatment effects for TMJ OA are limited. Understanding comprehensive molecular profiling of TMJ OA is an essential step in discovering new candidate target molecules that are potentially involved in the pathogenesis of TMJ OA. Hence, the aim of the present study was to investigate the role of the immune-related pathophysiology of TMJ OA by analyzing the transcriptional profiles of RNA from peripheral blood mononuclear cells (PBMC), which identify the differentially expressed genes (DEGs) of young females with TMJ OA.

## Materials and methods

### Participants

**Figure 1:**
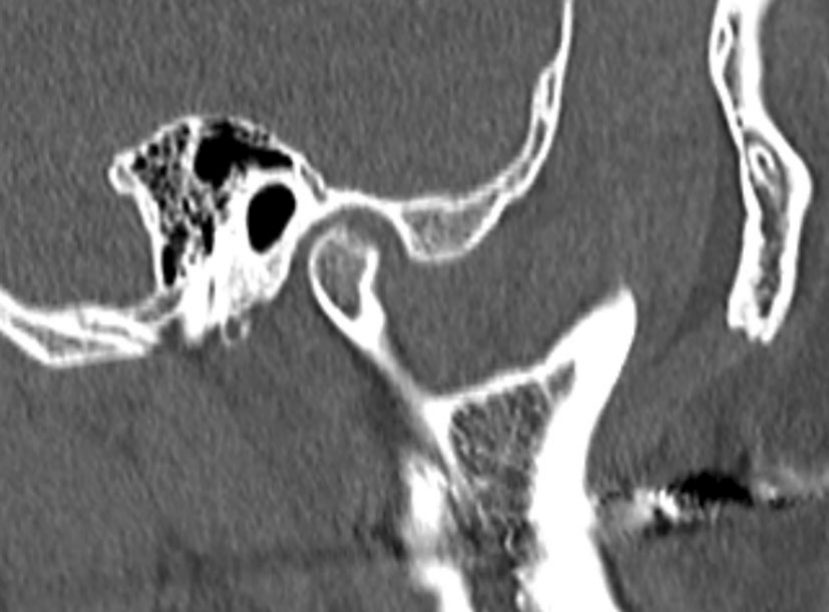
Sagittal cuts of CT which showed erosive osseous changes of TMJ.

In the present study, a total of 35 young females (mean age 19.7 ± 3.1 years; age range 15–25 years) were enrolled. Twenty-four female patients (mean age 19.3 ± 3.1 years; age range 15–25 years) with TMJ OA on at least one side of the condyle were consequently recruited from those who attended the TMD and Orofacial Pain Clinic at a university hospital from January, 2019 to November, 2019 (RNAOA). Eleven young females (mean age 20.5 ± 3.7 years; age range 15–25 years) without any sign of TMD and/or TMJ OA who voluntarily participated in the study served as control (CON). Patients with the following conditions were excluded: history of head and neck trauma prior to at least 6 months prior to study entry; autoimmune diseases, which could affect the systemic levels of inflammation including juvenile idiopathic arthritis, lupus erythematosus, ankylosing spondylitis; craniofacial anomalies; and neurodegenerative disorders. All participants in the RNAOA did not show any sign of capsule or myofascial pain in the orofacial area for at least 3 months before enrollment and had not been suffered from chronic pain which last for more than 3 months in other part of the body; hence the effect of pain condition on RNA transcription profiles could be excluded. Clinical parameters such as the degree of pain free opening and maximum unassisted opening as well as the duration of TMD symptoms including TMJ noise and difficulties in opening and/or closing the mouth were evaluated. Participants were diagnosed following the Diagnostic Criteria for Temporomandibular Disorder Axis I^20,21^. All female participants in the RNAOA showed erosive osseous bony changes on computed tomography (CT) images indicating the area of discontinuation of the cortical lining and adjacent bone (Fig. 1). The participants who showed TMJ condyles with osteophyte or combined changes with proliferation, deformed contour, and generalized sclerosis were excluded to rule out the patients with TMJ OA after bony remodeling. No participants showed positive sites on palpation on the temporalis and masseter muscles and TMJ capsules. The research protocol was approved by the Institutional Review Board of the University Hospital (AJIRB-MED-GEN-18-449). Informed consents were obtained from all participants or, if participants were under 18, from their parents and/or legal guardians. All methods were performed in accordance with the relevant ethical guidelines and regulations.

### RNA extraction from PBMC and RNA seq

To minimize the effect of circadian variation and menstruation cycle, peripheral blood was collected from all participants between 9:00 a.m. and 11:00 a.m. during their mid-luteal phases. For each individual, 3 ml of peripheral blood was put into a PAXgene Blood RNA tube vacutainer tube (Qiagen, New York, USA). PBMC from the venous blood from all participants were isolated using Ficolle-Paque PLUS (Sigma-Aldrich). TRIzol reagent (Thermo Fisher Scientific Invitrogen Inc., MA, USA) was used to isolate the total RNA from PBMC. Genomic DNA contamination was removed using RNase-free DNase I. The amount of total RNA was measured using NanoDrop 2000 (Thermo Fisher Scientific Invitrogen Inc., MA, USA) and the integrity and quality of total RNA samples were analyzed using an Agilent Technologies 2100 Bioanalyzer (Agilent Technologies, Inc., CA, USA). All samples passed quality control RNA integrity analysis (RIN ≥ 7). cDNA libraries were constructed using the TruSeq Stranded Total RNA Sample Preparation Kit (Illumina Inc, USA) according to the manufacturer’s protocol. To produce 100 bp paired-end reads, the total RNA was sequenced using the Illumina NovaSeq 6000 system (Macrogen, Seoul, Korea). After sequencing, the indexed samples were demultiplexed before the generation of FASTQ files for analysis and assessed by FastQC version 0.11.7.

Using HISAT2 version 2.1.0 with the best score matches reported for each read, all libraries were aligned to hg19 assembly of the human genome. The mapped reads were assembled using STRING Tie version 1.3.4d. Inter-gene expression comparisons were based on calculated fragments per kilobase of transcript per million (FPKM) mapped reads.

### Differential expression analysis of RNA-seq data

The expression level was normalized by calculating FPKM mapped reads. For DEG analysis, the value of log _2_ (fold change) were calculated. The DEGs with an adjusted *P* ≤ 0.05 and log _2_ (fold changes) ≤ -1 or ≥ 1 were determined. Using heatmap function, the hierarchical clustering of the expression profiles of detected DEGs was conducted.

### Bioinformatics analysis

Using online tool STRING analysis (http://string-db.org, version 11.0), gene ontology (GO) pathway enrichment analysis was conducted to compare gene transcription patterns between RNAOA and CON and assess the functional association between encoded proteins. False discovery rate (FDR) adjusted *P* values were calculated for each enriched biological pathway and the threshold was set to an adjusted *P* < 0.01. Gene set enrichment analysis (GSEA, version 4.0.3, http://www.gsea-msigdb.org) was used to compare gene dysregulation patterns in RNAOA with those in CON.

The threshold was set to an unadjusted *P* < 0.05 to fully explore the results by ingenuity pathway analysis (IPA; Ingenuity System Inc., Redwood City, CA, USA). Using IPA, biological processes, canonical pathways, and networks of analysis were analyzed. An enrichment score measures the overlap of observed and predicted gene sets. A z-score assesses the match of observed and predicted regulation patterns, serving as a predictor of the activation state of the identified molecule.

### Quantitative real time polymerase chain reaction validation

To validate the differential expression, gene expression levels were examined using quantitative real time polymerase chain reaction (qRT-PCR). qRT-PCR reactions were conducted via the Step ONE Plus (ABI, Life Technologies, CA, USA) using SYBR premix EX Taq II (Applied Biosystems, Foster City, CA, USA), and cDNA was synthesized using 1 μg of mRNA. The gene expression results were obtained using the formula 2^−(ΔCt)^, and the fold change was calculated by the formula 2^−(ΔΔCt)^. The qRT-PCR values were normalized using the average of the expression of the reference gene, GAPDH. Finally, the averaged fold ratios from the reference housekeeping gene were used as the relative mRNA level. Each experiment was conducted in triplicate. Two reactions, one without template and one without reverse transcriptase were also performed.

### Statistical analysis

The differences in demographic features and TMD characteristics between CON and RNAOA were evaluated using Mann–Whitney U tests. The differences in gene expression patterns were defined as fold changes. Statistical analysis was achieved by setting the change in gene expression threshold to an unadjusted *P* < 0.01 for encoded protein functional network analysis (FNA). The threshold was set to an unadjusted *P* < 0.05 to fully explore the results by IPA. To compare gene expression levels obtained by qRT-PCR between RNA OA and CON, Mann–Whitney U tests were utilized. The level of significance was set at *P* < 0.05.

## Results

### Demographic features and TMD symptoms in participants

The differences in age (*P* = 0.268) and body mass index (*P* = 0.740) did not show statistical significance between CON and RNAOA. Participants in RNAOA showed 16.4 ± 28.0 months of TMD symptom duration and the amount of pain free opening and maximum unassisted opening showed a significant difference between CON and RNAOA (*P* = 0.036) (Table 1).

**Table 1:** Demographic features and TMD characteristics of participants.

	CON (n = 11)	RNAOA (n = 24)	*P* value
Age	23.2 ± 11.4	19.3 ± 3.1	0.268
BMI	20.6 ± 2.2	20.5 ± 2.7	0.740
Duration of TMD symptoms (months)	0	16.4 ± 28.0	< 0.001**
Pain free opening (mm)	47.5 ± 4.7	42.8 ± 5.7	0.036*
Maximum unassisted opening (mm)	47.5 ± 4.7	42.8 ± 5.7	0.036*

Descriptive values are shown as mean ± SD.

Data obtained from Mann–Whitney U tests.

*P < 0.05, **P < 0.001 by Mann–Whitney U tests.

### Gene dysregulation in peripheral blood of patients with TMJ OA

**Figure. 2:**
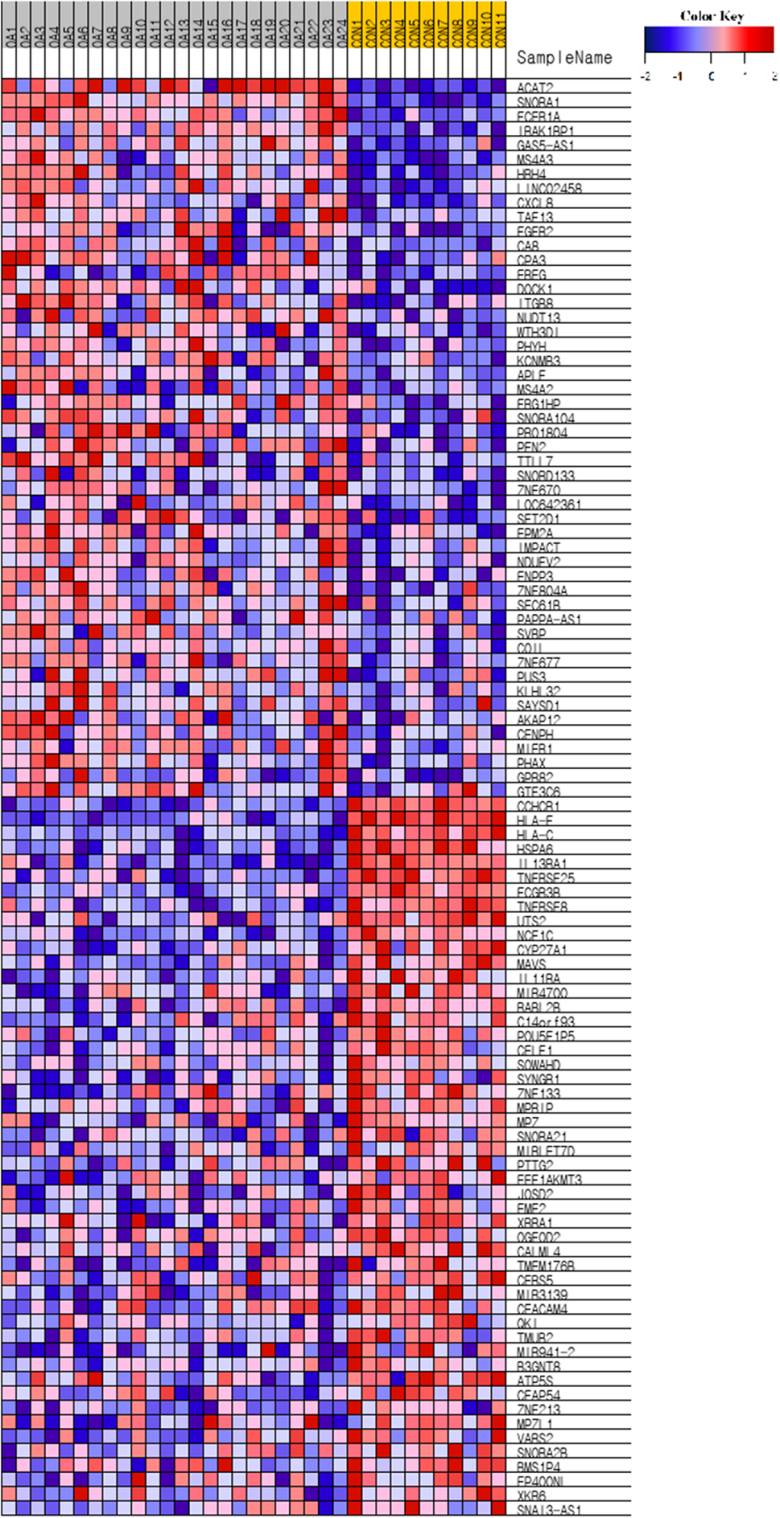
Heatmap demonstrating the expression patterns of DEGs between RNAOA and CON. The color key at the top indicates relative gene expression. Red and blue colors represent higher and lower gene expression levels, respectively. The figure was obtained from GSEA, version 4.0.3 (http://www.gsea-msigdb.org).

There were a total of 27,685 transcripts, and transcripts with an FPKM value less than 5 were excluded, leaving 12,788 transcripts to be analyzed. After excluding the following FPKM baseline gene, 440 genes were differentially expressed in the RNAOA compared with those in the CON (|fold change|> 1, *P* < 0.05). A total of 41 genes were expressed significantly differentially between RNAOA and CON with 17 genes upregulated and 24 downregulated in the RNAOA (log 2 (fold change) ≥ 1 or ≤ -1, *P* < 0.05) (Table 2). A heatmap using hierarchical clustering analysis showing the expression levels of each of these genes per individual was provided (obtained from GSEA, version 4.0.3) (Fig. 2). The genes with the highest fold change in RNAOA were FGFR2, EREG, WTH3DI, CXCL8, and LINC02458 and those with the lowest fold change were SYNGR1, CYP27A1, IL11RA, MAVS, and MIR941-2 (Table 2).

**Table 2:** Transcripts with altered gene expression in the RNAOA compared to CON.

Gene symbol	Gene name	Log_2_ (fold change)	*P* value
FGFR2	fibroblast growth factor receptor 2	1.51	0.020
EREG	epiregulin	1.33	0.029
WTH3DI	RAB6C-like	1.21	0.027
CXCL8	C-X-C motif chemokine ligand 8	1.19	0.048
LINC02458	long intergenic non-protein coding RNA 2458	1.18	0.041
SNORD133	small nucleolar RNA, C/D box 133	1.13	0.032
FCER1A	Fc fragment of IgE receptor Ia	1.13	0.036
MS4A3	membrane spanning 4-domains A3	1.12	0.042
RPL21	ribosomal protein L21	1.10	0.012
ITGB8	integrin subunit beta 8	1.08	0.004
GPR82	G protein-coupled receptor 82	1.08	0.049
LOC105377267	uncharacterized LOC105377267	1.07	0.024
SNORA1	small nucleolar RNA, H/ACA box 1	1.05	0.025
SNORA104	small nucleolar RNA, H/ACA box 104	1.05	0.020
MS4A2	membrane spanning 4-domains A2	1.04	0.017
HRH4	histamine receptor H4	1.03	0.029
ACAT2	acetyl-CoA acetyltransferase 2	1.03	0.039
GAS5-AS1	GAS5 antisense RNA 1	1.02	0.032
DOCK1	dedicator of cytokinesis 1	1.01	0.020
CA8	carbonic anhydrase 8	1.01	0.020
IRAK1BP1	interleukin 1 receptor associated kinase 1 binding protein 1	1.01	0.002
CPA3	carboxypeptidase A3	1.01	0.018
GTSF1	gametocyte specific factor 1	1.01	0.049
ZNF257	zinc finger protein 257	1.00	0.031
HLA-F	major histocompatibility complex, class I, F	-1.00	0.014
HSPA6	heat shock protein family A (Hsp70) member 6	-1.01	0.003
NCF1C	neutrophil cytosolic factor 1C pseudogene	-1.01	0.047
HLA-C	major histocompatibility complex, class I, C	-1.02	0.022
IL13RA1	interleukin 13 receptor subunit alpha 1	-1.02	0.034
TNFRSF8	TNF receptor superfamily member 8	-1.02	0.026
FCGR3B	Fc fragment of IgG receptor IIIb	-1.03	0.050
TMEM176B	transmembrane protein 176B	-1.03	0.047
MIR941-3	microRNA 941–3	-1.03	0.019
TNFRSF25	TNF receptor superfamily member 25	-1.03	0.039
CCHCR1	coiled-coil alpha-helical rod protein 1	-1.04	0.042
UTS2	urotensin 2	-1.04	0.043
SYNGR1	synaptogyrin 1	-1.06	0.030
CYP27A1	cytochrome P450 family 27 subfamily A member 1	-1.11	0.007
IL11RA	interleukin 11 receptor subunit alpha	-1.11	0.044
MAVS	mitochondrial antiviral signaling protein	-1.14	0.029
MIR941-2	microRNA 941–2	-1.46	0.031

### Functional network analysis

**Figure 3:**
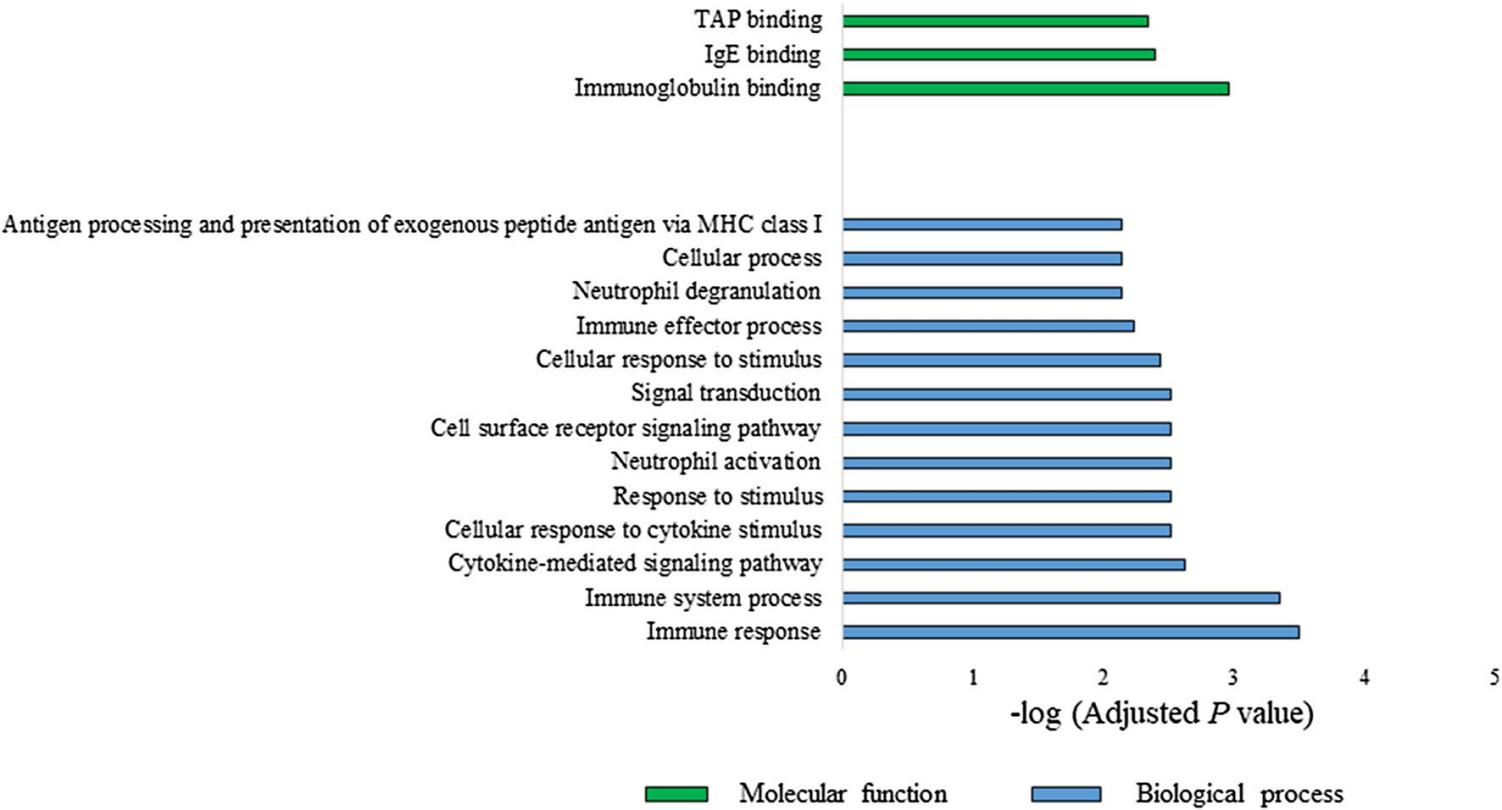
GO pathway enrichment analysis of dysregulated genes in peripheral blood of young females with TMJ OA. A total of 16 GO terms (*P* < 0.01) including 3 molecular and 13 biological terms were annotated using the GO function of molecular function and biological process.

**Figure 4:**
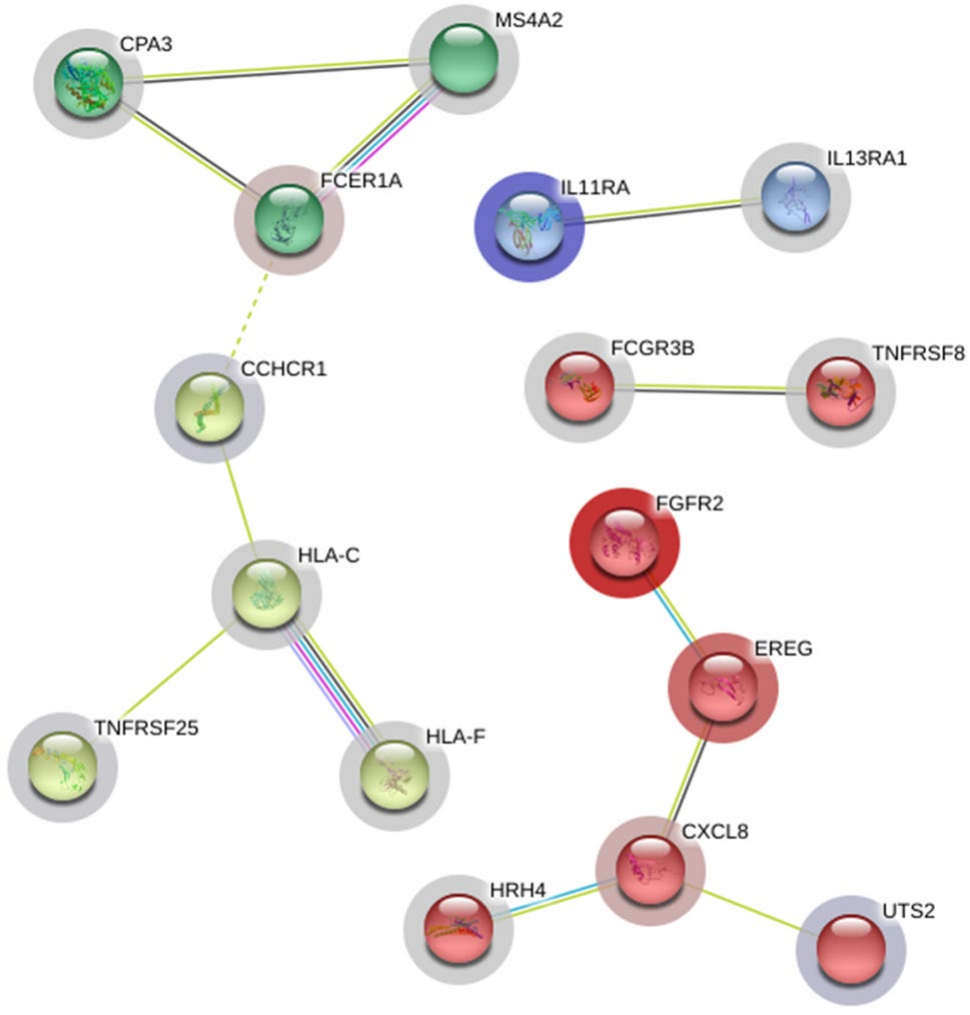
Four clusters of strong functional associations with significant levels of network enrichment. The figure was obtained from STRING portal (http://string-db.org, version 11.0).

The FNA of proteins encoded by differentially expressed gene transcripts (n = 41, *P* < 0.05) were assessed using the STRING portal (http://string-db.org, version 11.0). A total of 16 GO terms (*P* < 0.01) including three molecular and 13 biological terms were annotated by the GO function of molecular function and biological process (Fig. 3). Four clusters of strong functional associations with significant levels of network enrichment (*P* = 0.000821) were observed: (1) HLA-C, HLA-F, TNFRSF25, CCHCR1, FCER1A, MS4A2, and CPA3, (2) FGFR2, EREG, CXCL8, and HRH4, and UTS2 (3) FCGR3B and TNFRSF8, and (4) IL11RA and IL13RA1 (Fig. 4).

### Pathway analysis using IPA

IPA was carried out on the transcriptome dataset with significance set at *P* < 0.05 and |log 2 (fold change)| > 1. IPA identified 20 annotated categories of diseases and functions that were significantly upregulated (z-score > 2.0) and included 13 of the DEGs from the study. The 5 top canonical pathways were associated with the role of natural killer cells, communication between innate and adaptive immune cells, and T cell signaling (Table 3). Nine genes were involved in five top canonical pathways namely, FCGR3B, HLA-C, HLA-F, HSPA-6, CXCL8, ITGB8, FCER1A, IL11RA, and IL13RA1. Hence, these pathways reinforce significant immune and inflammatory dysregulation occurring in the pathogenesis of TMJ OA.

**Table 3:** Annotated categories of diseases and functions from IPA.

Ingenuity canonical pathways	Z-score	Molecules
Natural killer cell signaling	3.63	FCGR3B, HLA-C, HLA-F, HSPA6
Communication between innate and adaptive immune cells	3.36	CXCL8, HLA-C, HLA-F
Virus entry via endocytic pathways	3.23	HLA-C, HLA-F, ITGB8
White adipose tissue browning pathway	2.99	FCER1A, FGFR2, MS4A2
STAT3 pathway	2.93	FGFR2, IL11RA, IL13RA1
Cytotoxic T lymphocyte-mediated apoptosis of target cells	2.9	HLA-C, HLA-F
Antigen presentation pathway	2.78	HLA-C, HLA-F
Tec kinase signaling	2.69	FCER1A, MS4A2, TNFRSF25
Graft-versus-Host disease signaling	2.61	HLA-C, HLA-F
Autoimmune thyroid disease signaling	2.59	HLA-C, HLA-F
Role of NFAT in regulation of the immune response	2.57	FCER1A, FCGR3B, MS4A2
Dendritic cell maturation	2.56	FCGR3B, HLA-C, HLA-F
Systemic lupus erythematosus signaling	2.28	FCGR3B, HLA-C, HLA-F
Cardiac hypertrophy signaling (enhanced)	2.19	CXCL8, FGFR2, IL11RA, IL13RA1
Allograft rejection signaling	2.11	HLA-C, HLA-F
CTLA4 signaling in cytotoxic T lymphocytes	2.08	HLA-C, HLA-F
Crosstalk between dendritic cells and natural killer cells	2.08	HLA-C, HLA-F
Protein ubiquitination pathway	2.07	HLA-C, HLA-F, HSPA6
OX40 signaling pathway	2.07	HLA-C, HLA-F
Fcγ receptor-mediated phagocytosis in macrophages and monocytes	2.04	DOCK1, FCGR3B

### qRT-PCR

**Figure 5:**
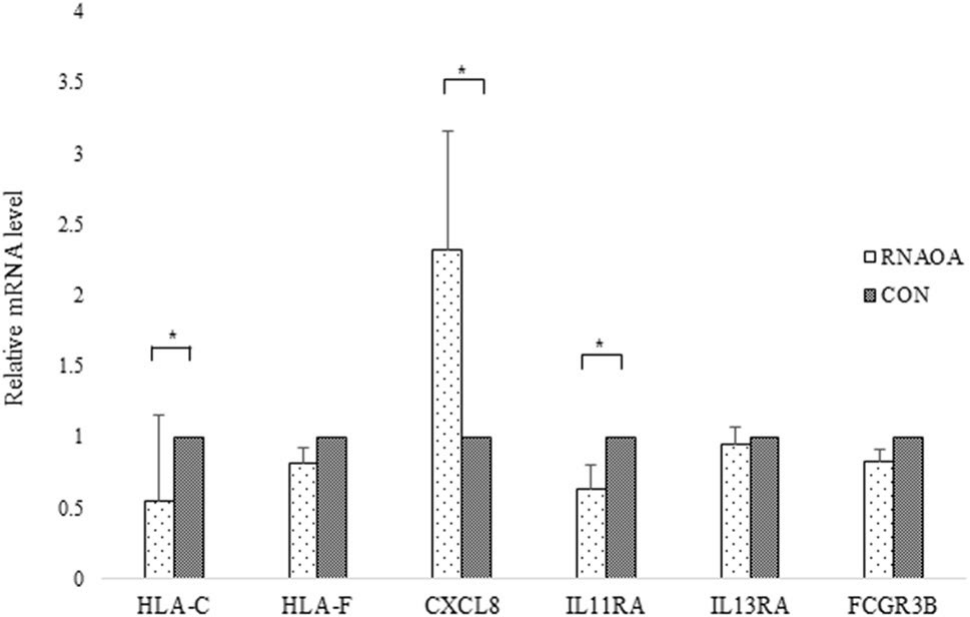
Validation of RNA-seq results using qRT-PCR. Normalized fold expressions of mRNA are shown. Error bar represents mean ± standard error. Mann–Whitney U tests were used to compare gene expression levels (*P < 0.05).

The gene expression patterns of 6 hub genes, including HLA-C, HLA-F, CXCL8, IL11RA, IL13RA1, and FCGR3B transcripts which showed significant results in both GO enrichment analysis and IPA were tested via qRT-PCR. Significant differences were observed in HLA-C (*P* = 0.030), CXCL8 (*P* = 0.022), and IL11RA transcripts (*P* = 0.018) (Fig. 5).

## Discussion

Early onset of diseases, especially during pubertal phases and age of early 20 s is a unique feature of the TMJ OA^2,3^. The progression of the TMJ OA may accompany compromised masticatory function and altered craniofacial morphology, which could affect an individual’s quality of life^22–24^. However, to date, the exact pathophysiology of TMJ OA particularly in young patients and clear molecular mechanisms of the development of TMJ OA have not yet been elucidated, so far. Immune dysfunction has been considered as one of the main etiological factors which may result in bony destruction in patients with OA from other joints^6–9,12,13^, but studies which clearly elucidate the immunological background of TMJ OA especially in young patients are sparse. RNA-seq is an effective tool for clarifying molecular mechanisms in genetic levels and could provide the novel therapeutic targets involved in a certain condition^19^. To the best of the knowledge, no study ever attempted to reveal the immune related etiology of TMJ OA using RNA-seq technology. Consequently, the aim of the present study was to investigate the role of immune dysfunction by analyzing transcriptional profiles of PBMC and search for new therapeutic targets for management of TMJ OA in young patients.

Declined acquired immune responses accompanied by increased autoreactivity have been detected in the elderly^25^ and relationships between this altered innate immune function, T cell and B cell responses, and cartilage and bone degradation have been reported^26,27^. Aforementioned results from IPA demonstrated associations among various autoimmune disorders, including autoimmune thyroid disease, systemic lupus erythematosus (SLE), and occurrence of TMJ OA. Previous studies already have mentioned the high prevalence of temporomandibular disorders in patients with autoimmune thyroid disorders or SLE^28–30^. Even though severe condylar resorption in patients with rheumatoid arthritis or juvenile idiopathic arthritis has been detected^31^, few reports ever mentioned the bony changes of condyles in other autoimmune diseases such as autoimmune thyroid disorders or SLE. Because patients with autoimmune disorders were excluded from the present study, clear associations between autoimmune thyroid disorders or SLE and TMJ OA could not be clarified. Even though, previous studies which dealt with the associations between innate immunity and OA progression focused on increased autoreactivity related to the aging process, the role of altered innate immune function in bony destruction of TMJ condyles even in young females could be assumed through this study.

Accumulating evidence suggested the involvement of inflammatory and immune responses in the pathogenesis of OA^32^. The results from the present study demonstrated the increased expression levels of chemokine (C-X-C motif) ligand 8 (CXCL8), the involvement of signal transducers and activators of transcription 3 (STAT3) pathway, and cytokine mediated immune response in the development of TMJ OA in young females. Interleukin-8/CXCL8 (IL-8/CXCL8) has been found to be an attractant for neutrophils and a population of lymphocytes^33^. The increased level of IL-8 in synovial fluid from the knee OA and TMD patients with disc displacement has been reported^34–37^, but the mechanisms of IL-8 in subchondral bone changes in TMJ have not been clearly revealed. One study has reported elevated serum levels of CXCL8 and expression levels of STAT3 in knee OA patients^38^. This study suggested that CXCL8 may inactivate the mitosis of chondrocytes and further aggravate OA progression and indirectly induce the Janus kinase (JAK)/STAT3 signaling in chondrocytes. Although the specific molecular mechanisms of CXCL8 and STAT3 could not be revealed in the present results, the associations between CXCL8, STAT3 signaling, and subchondral bony destruction of TMJ condyles in young females could be suspected.

The peripheral blood of patients with OA have been analyzed and revealed that patients with OA have shown altered levels of CD8 + T cells and more cytotoxic profiles in comparison with healthy controls^11,39–41^. One animal study suggested the molecular mechanisms of the role of CD8 + T cells in the OA process that CD8 + T cells were activated once OA had been initiated and cartilage degeneration occurred more slowly in CD8 + T cell knockout mice than in wild-type^42^. The results from this study also showed the relationships between the occurrence of TMJ OA and T cell activity through increased expression levels of genes related to Tec kinase signaling, nuclear factor of activated T cells (NFAT) regulation, and OX40 signaling. Furthermore, cytotoxic T lymphocyte mediated apoptosis and CTLA4 signaling pathway which have an association with cytotoxic T cell function, seem to have roles in the incidence of TMJ OA. A previous study has focused on the role of T helper cell in synovial fluid in the subchondral bony changes in TMJ OA patients^16^, but no study ever attempted to clarify altered cytotoxic T cell activities in blood in patients with TMJ OA.

Due to cross-sectional study design, the causal relationships between altered RNA transcriptional profiles and development of TMJ OA could not be derived from this study. The altered transcriptional profile would be result from TMJ bony destruction, and also abnormal immune-modulation function could be one of the main etiological factors of the TMJ OA. Because TMJs are small peripheral joints, the bony changes of the TMJs may not induce prominent changes in the systemic transcriptional profiles in the peripheral blood. However, regarding the statistically significant differences in the selected pathway and detected genes and sample number over 30, those differences could not be results just from chance.

To the best of our knowledge, the present study is the first study which attempted to reveal the genetic and molecular background of TMJ OA in young patients using the RNA-seq technology. Several previous reports which dealt with the systemic immune dysfunction and the development of OA have focused on the role of the aging process and the senescence of immune cells. In addition, most of the studies which analyzed the relationships between levels of the immune-modulating agents in blood and OA dealt with weight bearing axial joints such as the knee and hip not with peripheral joints. Aforementioned results showed that altered systemic immune function could have a role in the development of arthritic conditions even in young patients with OA from small peripheral joints such as TMJ. However, the present study still has several limitations, first of all, RNA-seq was conducted using only PBMC, not using the synovial tissue. However, because TMJ is small joint, synovial fluid collecting process which accompanies with puncturing process inevitably causes iatrogenic inflammation in the small joint space and this may affect the RNA transcriptional profiles. Furthermore, the aim of this study was to figure out the immune-related pathophysiology of TMJ OA in young females, so adopting peripheral blood would be essential to reveal the purpose of this study. Secondly, only female participants were included. Even though previous studies confirmed the female preponderance of TMJ OA, the absence of male participants would limit the understanding of the genetic etiology of the TMJ OA. Thirdly, owing to the cross-sectional characteristics of the study, the information regarding the changes in the transcriptome profiles in accordance with the progression or recovery of TMJ OA could not be provided. Finally, the lack of control with inflammatory arthritis including juvenile idiopathic arthritis and rheumatoid arthritis could give limited information. However, no study which tried to reveal the immune-related pathophysiology of the TMJ OA based on transcriptional profiles of the peripheral blood have been investigated. Future prospective RNA-seq studies with large samples including both male and female participants and another control with inflammatory arthritis would be necessary.

In conclusion, in the present study, young female TMJ OA patients showed alterations in the blood immune cell expression and some of the changes may reflect inflammation, autoreactivity, and altered T cell functions. For proper management and successful treatment of TMJ OA in young females, future research regarding immune-modulation based therapy would be warranted.

## Data Availability

The datasets used and/or analyzed during the current study are available from the corresponding author on reasonable request.
